# Evaluation of Color and Pigment Changes in Tomato after 1-Methylcyclopropene (1-MCP) Treatment

**DOI:** 10.3390/s24082426

**Published:** 2024-04-10

**Authors:** Zsuzsanna Horváth-Mezőfi, László Baranyai, Lien Le Phuong Nguyen, Mai Sao Dam, Nga Thi Thanh Ha, Mónika Göb, Zoltán Sasvár, Tamás Csurka, Tamás Zsom, Géza Hitka

**Affiliations:** 1 Institute of Food Science and Technology, Hungarian University of Agriculture and Life Sciences (MATE), H-1118 Budapest, Hungary; horvath-mezofi.zsuzsanna@uni-mate.hu (Z.H.-M.); baranyai.laszlo@uni-mate.hu (L.B.); nguyen.le.phuong.lien@uni-mate.hu (L.L.P.N.); ngahtt@hufi.edu.vn (N.T.T.H.); gob.monika@phd.uni-mate.hu (M.G.); sasvar.zoltan.arpad@phd.uni-mate.hu (Z.S.); csurka.tamas@uni-mate.hu (T.C.);; 2 Industrial University of Ho Chi Minh City, Ho Chi Minh 700000, Vietnam; damsaomai@iuh.edu.vn; 3 Faculty of Food Science and Technology, Ho Chi Minh City University of Industry and Trade, Ho Chi Minh 700000, Vietnam

**Keywords:** tomato, 1-MCP, chlorophyll fluorescence, image processing, quality

## Abstract

The Polar Qualification System (PQS) was applied on hue spectra fingerprinting to describe color changes in tomato during storage. The cultivar ‘Pitenza’ was harvested at six different maturity stages, and half of the samples were subjected to gaseous 1-methylcyclopropene (1-MCP) treatment. Reference color parameters were recorded with a vision system colorimeter instrument, and the fruit pigment concentration was assessed with the DA-index^®^. Additionally, acoustic firmness (Stiffness) was measured. All acquired reference parameters were used to grade fruit in the supply chain. The applied 1-MCP treatments were used to control the ripening of climacteric horticultural produce. Both the DA-index^®^ and stiffness values, presented as chlorophyll concentration and acoustic firmness, showed significant differences among maturity stages and treated and control samples and in their kinetics during storage. The machine vision parameter PQS-X was significantly affected by 1-MCP treatment (F = 10.18, *p* < 0.01), while PQS-Y was primarily affected by storage time (F = 18.18, *p* < 0.01) and maturity stage (F = 11.15, *p* < 0.01). A significant correlation was achieved for acoustic firmness with normalized color (r > 0.78) and PQS-Y (r > 0.80), as well as for the DA-index^®^ (r > 0.9). The observed color changes agreed with the reference measurements. The significant statistical effect on the PQS coordinates suggests that hue spectra fingerprinting with this data compression technique is suitable for quality assessment based on color.

## 1. Introduction

Tomato (*Solanum lycopersicum* L.) is an important horticultural product due to its attractive red color and nutritional value, containing high amounts of antioxidants [1]. Tomato has a high consumption level all year round. However, a limitation of tomato in the supply chain is rapid postharvest ripening, which results in significant quality and market value losses. Thus, treatments and practices extending the shelf life and maintaining the quality of tomato after harvest are very necessary.

The application of 1-methylcyclopropene (1-MCP) successfully controls the ripening of fruits and vegetables after harvest by inhibiting the negative effects of ethylene. There have been many reports about the role of 1-MCP in delaying the ripening and retaining the texture, taste and appearance of fruits and vegetables [2,3,4]. The 1-MCP treatment was shown to significantly delay the ripening of tomato [5]; however, the level of efficacy depends on the physiological stages of tomato [6,7].

The color of tomato changes during fruit development and ripening; therefore, color measurement is a popular non-destructive technique in its quality assessment [8]. The standard CIE Lab color parameters are typically measured with colorimeters (or chromameters), and the change in the a* color characteristic value, representing the color change along the green–red axis of the color space diagram, is used for tomatoes. It was found that on-vine and detached fruit behave differently, and especially sensitively react to storage temperature. Tomatoes kept at high temperature have increased β-carotene (orange) and decreased lycopene (red) content [1]. Color is the primary quality parameter in cold storage below 8 °C, while firmness becomes more important above 13 °C [9]. The USDA color grades [10] of mature green (green, breaker), intermediate (turning, pink) and advanced (light red, red) describe ripeness, and this classification strongly correlates with internal quality attributes. Regarding color parameters, both colorimeter and machine-vision-measured hue angle and a*/b* achieved a strong linear relationship with total soluble solids (TSSs) for the tomato Master 100 hybrid [11]. The comparison of different tomato genotypes also revealed the difference in the pigmentation (chlorophylls, carotenoids, anthocyanins) of genotypes, which may result in black and purple colors as well [12]. It was found that native genotypes of different colors are rich in functional compounds, such as tocopherols, flavonoids and vitamin C. A digital phenotyping tool has been introduced to measure tomato color and its uniformity based on machine vision [13,14]. This Tomato Analyzer software (Tomato Analyzer ver. 4.0, Athens, GA, USA) saves the red, green and blue color components besides luminosity, converted a* and b*, hue angle and chroma. It was observed that measured red, green and blue intensity values differ significantly when a color difference is detected.

The results of several researchers and research groups [15,16] show that the photosynthetic activity of horticultural crops containing chlorophyll, i.e., freshness/ripeness, quality properties and shelf life, can be determined non-destructively, quickly, easily and relatively cheaply by chlorophyll fluorescence spectroscopy. According to Kasampalis et al. [17], chlorophyll fluorescence measurements can be used innovatively and at least as efficiently and reliably as tristimulus colorimetry to classify tomatoes according to maturity. Ripe red tomatoes with different fluorescence values could be further subclassified. Both visible (Vis) and near-infrared (NIR) spectroscopy are non-destructive methods that can be used to describe chemical properties (color content, moisture, carbohydrate, water-soluble solids, starch, lycopene content, pH), as well as ripening and spoilage processes [18,19]. 

**Figure 1:**
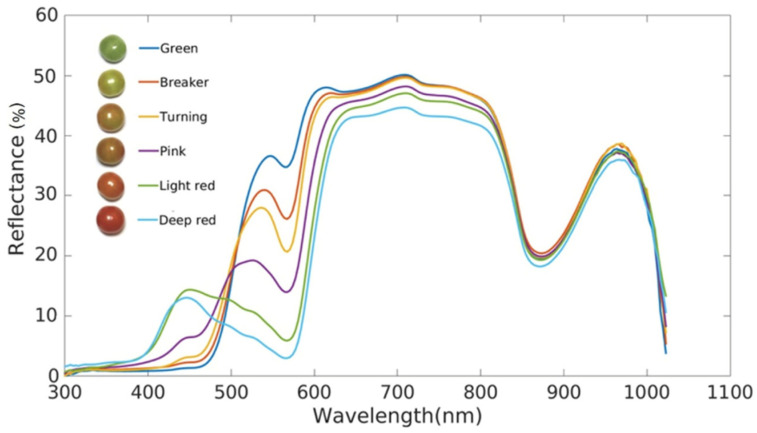
Reflectance of tomatoes at different stages of ripeness [20].

Li et al. [20] investigated the relationship between reflected light and wavelength using reflectance spectroscopy for tomato at different stages of ripening, rather than absorbance and wavelength (Figure 1). Using this method, they were able to distinguish tomatoes at different stages of ripeness in the range of 400–1100 nm.

The use of the non-destructive acoustic firmness measurement method is widespread, as it is excellent for testing the internal hardness and global firmness of spherical, homogeneous products such as apples, peaches, plums, pears, melons and tomatoes. The method relies on the propagation of mechanical waves. It is based on the acoustic sound response method, which is the study of the natural vibration caused by mechanical excitation (mechanical or manual low-pulse impact or vibration). Thus, the acoustic response (natural vibration) of a sample to the excitation gives comprehensive information about the hardness of the crop, while the resonance frequency carries information about the texture and its quality. The value of the resonance frequency is influenced by the hardness of the sample and its weight and shape, but not by the speed of the impact [21,22]. In vivo measurements on tomatoes found that the acoustic firmness coefficient of the tomato berry decreases as the ripening process progresses. The temporal variation in softness is not uniform, increasing significantly when the berry changes color from green to red [22].

In addition, as a short summary, the most common methods used to monitor tomato ripening and their advantages and disadvantages are presented in Table 1.

The aim of this study was to investigate the feasibility of the digital image processing method (within the hue spectra fingerprinting with PQS data compression technique) for monitoring tomato ripening and to evaluate the effect of the 1-MCP SmartFresh^TM^ anti-ripening treatment on the maintenance of tomato quality during postharvest storage.

## 2. Materials and Methods

### 2.1. Materials

**Figure 2:**
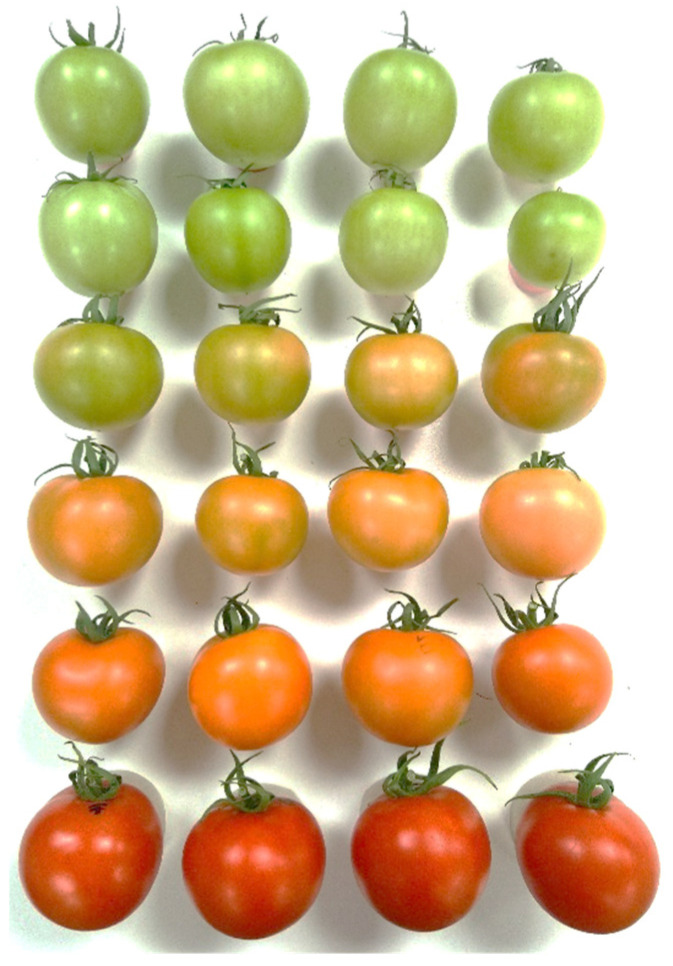
Six maturity stages selected after sorting tomatoes by color based on CTIFL scale [24].

The tested tomatoes were freshly harvested according to color-related different maturity stages, as shown in Figure 2, in Budapest, Hungary. Samples belonged to the Pitenza cultivar. Pitenza is a cluster tomato hybrid, which is widely used all over the world. The average mass of the berries is 100–120 g; the shape is rounded and dark red when ripe for consumption. The cultivar has excellent storage qualities and good yields under different growing conditions. It is one of the few varieties that can produce good quality cluster tomatoes even during the winter [23].

After delivery and color classification, the tomatoes were assigned into 6 different maturity groups (Figure 2). The color classification was based on the internationally accepted CTIFL (Centre Technique Interprofesionnel des Fruits et Légumes) scale of 1 to 12 [24], where 1 indicates tomatoes that are ripe green and 12 indicates tomatoes that are fully ripe for consumption. The selected maturity stages are listed in Table 2.

### 2.2. Implementation of Treatment

The applied anti-ripening treatment was the SmartFresh^TM^ (SF) treatment, manufactured and marketed by AgroFresh Inc. (Philadelphia, PA, USA). The maturity regulator agent used was SmartFresh^TM^ Protabs (Licence number: 04.2/1181-3/2017); the active ingredient of this agent is 2% 1-MCP gas (CAS registration number: 3100-04-7). The manufacturer’s recommendation for tomato treatment time is 12–24 h. After color grading and labeling, half of the samples (20 fruits per group) were randomly selected and treated for 12 h with SmartFresh^TM^, except for the red (F) group, which was composed of fully ripe tomatoes as the absolute control group. The treatment was carried out in an airtight plastic box (V = 0.5 m^3^) equipped with an internal fan, with a calculated amount of 1-MCP according to the manufacturer’s recommendation. The concentration of 1-MCP gas during treatment was approximately 625 ppb. The box was placed in a cooler at 15 °C for the duration of the treatment, while the control samples were stored at 15 °C. After the treatment was completed, the treated and control samples were stored in the same refrigerator at 15 °C for 2 weeks.

### 2.3. Color Measurements

The surface color changes in tomatoes were monitored using a portable Konica Minolta CR-400 colorimeter (Minolta Europe GmbH, Langenhagen, Germany). The instrument measures the CIE Lab color characteristics (L*, a*, b*, C* and h°). The measurements were carried out using the 8 mm diameter head of the instrument calibrated to the corresponding white etalon (No: 15033034; Y = 93.7, x = 3131, y = 3191) before starting the measurement. Measurements were taken at 2 points on each sample, along the maximum diameter of the tomato in a perpendicular position to the longitudinal axis, on two opposite sides of the berry.

### 2.4. Chlorophyll-Content-Related Maturity Stage Measurements

Changes in the chlorophyll-content-related maturity of the tomatoes and during storage were monitored using the Vis/NIR DA-meter^®^ type FRM01-F (Sintéleia s.r.l., Bologna, Italy). The chlorophyll content of the plant tissue was used to monitor maturity, which was determined by the instrument based on absorbance properties shown in Equation (1). Data acquisition was carried out by measuring the difference in absorbance between two different wavelengths. One of the measured wavelengths was the absorption peak of chlorophyll-a (670 and 720 nm), and the other was the reference wavelength during maturation to ensure minimum absorption. The chlorophyll content was determined using the DA-index^®^ ranging between 0 and 5 with an accuracy of 0.01. The higher the DA-index^®^ is, the more green the plant material is, together with the higher level of photosynthetically active chlorophyll content. The value of the DA-index^®^ decreases significantly as ripening progresses compared to the harvest stage [25].

DA-index^®^ = I_AD_ = A_670nm_ − A_720nm_ (1)

### 2.5. Image Processing

Color digital images with 3 × 8 bit/pixel were captured and saved in JPEG (Joint Photographic Experts Group) format. Fifteen samples were placed in front of the camera at the same time, 50 cm below the lenses. Tomato samples were illuminated by the laboratory ceiling-mounted, commercially available LED light panels with 3000 K color temperature. A white background was used, which additionally served as a color reference. Pictures were normalized to have the same white background so that potential fluctuations in illumination color could be managed.

The hue spectrum [26,27] was calculated for each picture with slight modification, as saturation was scaled in the range of 0–100%. Since saturations were summarized for the observed hue angles, the result was not a simple histogram of colors, but the colors were weighted with their vividness. Important colors appeared with peaks, and those peaks may have changed shape and position during ripening. The gray-scaled, low-saturation background and surface reflections were automatically ignored in the analysis. The resulting hue spectra were compressed with the Polar Qualification System (PQS) surface method [28]. It transformed the spectra into polar data, and the gravity point of the visible graph was computed. The gravity point location was expected to change with different spectra shapes caused by color changes.

Reference color data as averages of red, green and blue color components and their normalized values were calculated as well. Normalization removes intensity differences; therefore, these indices only reflected changes in color. The normalized values were computed by following Equation (2).

(2) $$R_{N}=\frac{R}{R+G+B}\;,G_{N}=\frac{G}{R+G+B},\;B_{N}=\frac{B}{R+G+B}$$

where R, G and B represent the average intensity of red, green and blue color components on the surface, and R_N_, G_N_ and B_N_ are the normalized values. High-saturation pixels were segmented as regions of interest (ROIs) by simple thresholding. The threshold was calculated based on the saturation histogram.

### 2.6. Acoustic Firmness Measurements

Changes in the texture of the samples were monitored with an Aweta AFS desktop firmness meter (AWETA AFS Desktop System, DTF V0.0.0.105, AWETA BV., Pijnacker, The Netherlands) connected to a computer. This instrument can be used for the acoustic and impact texture measurement of several types of crops with spherical or nearly spherical shapes. The acoustic firmness coefficient of a sample can be determined by the following formula (Equation (3)) based on De Ketelaere et al. [29]:

S = f^2^ × m^2/3^ (3)

where the terms are defined as follows:

S—acoustic firmness coefficient (g^2/3^s^−2^ or Hz^2^g^2/3^);
f—resonance frequency (Hz);
m—weight of the tested crop (g).

### 2.7. Data Analysis

Data were collected, pre-processed and plotted in graphs using routines in Microsoft^®^ Excel^®^ (version 2401). Statistical analysis was performed using SPSS (version 29.0.1.0, Armonk, NY, USA, 2022). A two-way analysis of variance (ANOVA) test was performed to detect significant effects. In addition to the main effects of treatment and maturity status, we included interaction effects in the models. The homogeneity of variances was assessed using the Levene test. Following the ANOVA test, parameters of homogeneous variances were further analyzed using the Tukey HSD post hoc test, while parameters of inhomogeneous variances were further analyzed using the non-parametric Games–Howell test. Significant differences were defined at *p* < 0.05. The relationship between the measured parameters was evaluated using both Pearson’s and Spearman’s rank correlation, due to the expected nonlinear behavior of pigment concentration. Data were plotted in graphs with mean ± standard deviation.

Collected pictures were processed with Scilab (version 6.1.1, Dassault Systèmes, Vélizy-Villacoublay, France) and the Image Processing and Computer Vision toolbox (IPCV 4.1.2).

## 3. Results

### 3.1. Changes in Surface Color

During the experiment, the L*, a* and b* color parameters were also determined with the Konica-Minolta colorimeter, but the L* lightness factor and the b* blue–yellow color factor did not provide relevant information on the color change in tomatoes, so these data are not reported in this article. As the surface color of the tomato shifts from green to red as it ripens, the red–green color parameter a* provided the most relevant information.

**Figure 3:**
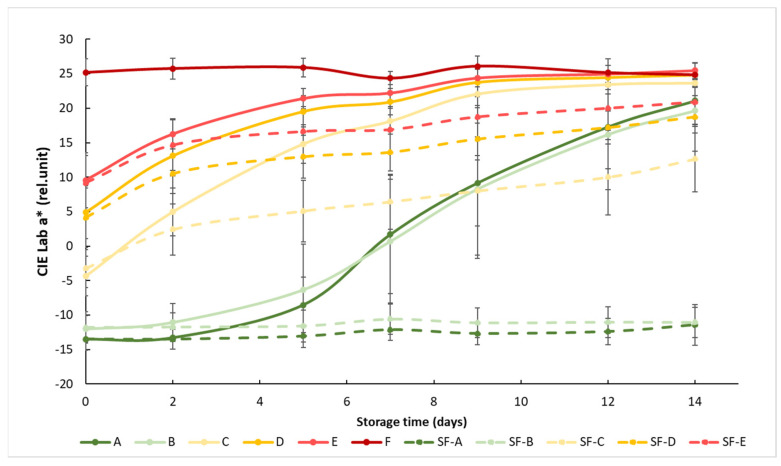
Changes in a* color values during storage in SmartFresh^TM^−treated and untreated tomatoes for all ripening stages. Data are presented with mean ± standard deviation. Colors ranging from dark green (A) to red (F) represent the different tomato ripening stages. Markings ranging from SF−A to SF−E represent sample groups subjected to SmartFresh^TM^ (SF) treatment.

At the beginning of the measurement, the obtained a* showed a clear separation between the different maturity stages, but this difference decreased as the storage time progressed, since the samples matured closer to the color of the fully mature reference group F. The effect of the SmartFresh^TM^ anti-ripening treatment was visible from day 2, but the difference between the treated and untreated groups was significant after about 1 week (Figure 3). The treatment was most effective for tomato groups A (mature green) and B (breaker), because the color of the treated samples in these two groups remained almost unchanged until the end of the two-week storage period. The treatment was also effective in the C, D and E ripening stages, but these tomatoes continued to change their color during storage, only at a slower rate than the control samples.

Statistical analysis showed that both treatment and maturity status had significant effects on the change in the red–green color characteristics of the samples (Table 3). Treatment significantly affected the color of the samples at all maturity stages. For the control groups, early (group A and B) and advanced (group D and E) maturity stages behaved similarly, while they were different from others, including the samples in the turning maturity stage. For the SmartFresh^TM^−treated samples, groups A and B were not significantly different from each other, while groups C, D and E were significantly different from all other groups. In general, Figure 3 shows the initial maturity-stage-dependent effect of SmartFresh^TM^ as it provided the postharvest ripening suppression of table tomatoes stored at ambient storage temperatures of 15 °C. 

### 3.2. Changes in DA-Index^®^

**Figure 4:**
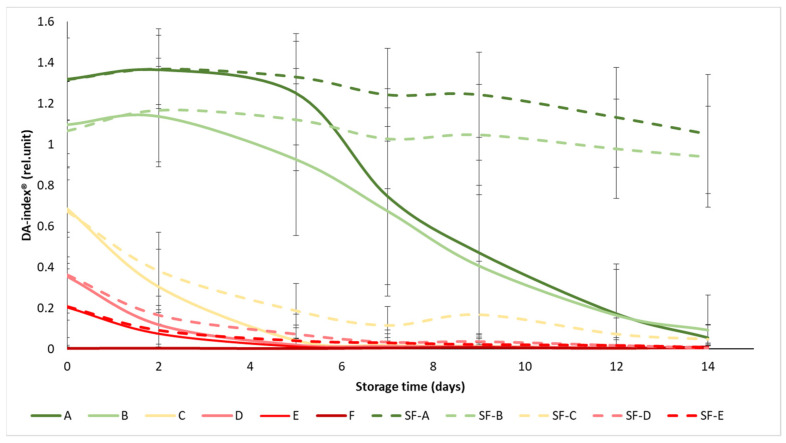
Changes in DA-index^®^ during storage in SmartFresh^TM^−treated and untreated tomatoes for all ripening stages. Data are presented with mean ± standard deviation. Colors ranging from dark green (A) to red (F) represent the different tomato ripening stages. Markings ranging from SF−A to SF−E represent sample groups subjected to SmartFresh^TM^ (SF) treatment.

The initial values of the DA-index^®^ ranged from 1.3 to 0.003, while all values dropped below 1.05 at the end of the experiment (Figure 4). The ripening stages were well separated at the beginning of the experiment, especially in the case of the A and B maturity stages, where the difference between SmartFresh^TM^−treated and control samples became apparent as the experiment progressed, from day 5 onwards. In maturity stage C (turning), the difference in treatment was also visible, but not significant, while in groups D and E, the treatment did not provide any significant benefit in terms of chlorophyll fluorescence, with the DA-index^®^ of the samples decreasing to almost zero from day 7, regardless of treatment. These results also support the previous statement that the mature green (A) and the breaker (B) tomatoes gained the greatest benefit from SmartFresh^TM^ treatment, as these groups showed minimal reductions in chlorophyll content (and thus in maturity change) by the end of the two-week storage period. 

Similarly to Figure 4, the statistical analysis also confirms that the treatment had a significant effect on the DA-index^®^ only in the mature green (A), breaker (B) and turning (C) groups (Table 4). In terms of maturity status, the mature green (A) and breaker (B) groups in the control group were significantly different from the turning (C), pink (D) and light red (E) groups, while the latter three were not significantly different from each other. For the treated samples, groups A and B were also not different from each other, as were D and E, while group C was different from the overall group.

### 3.3. Changes in Texture

**Figure 5:**
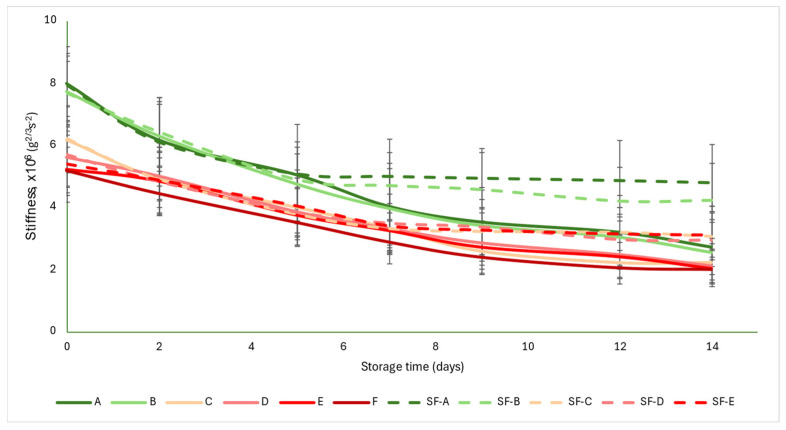
Changes in acoustic firmness during storage in SmartFresh^TM^−treated and untreated tomatoes for all ripening stages. Data are presented with mean ± standard deviation. Colors ranging from dark green (A) to red (F) represent the different tomato ripening stages. Markings ranging from SF−A to SF−E represent groups subjected to SmartFresh^TM^ (SF) treatment.

The acoustic firmness coefficient (S) was not as strongly differentiated for each group as the a* color parameter was. The initial values were between 5 and 8 g^2/3^ s^−2^. As the ripening progressed, the values showed monotonous decreases, with the control group values falling below 3 g^2/3^ s^−2^ by the end of the storage period, while the SmartFresh^TM^−treated samples averaged between 2.94 and 4.2 g^2/3^ s^−2^. From about the seventh day onwards, the difference between the control and treated samples became apparent and the difference increased slightly with time. The data obtained here also support the conclusion that the treatment was most effective in the mature green (A) and breaker (B) groups, where the difference between the control and treated samples was the largest at the end of the storage period (Figure 5).

Figure 5 is supported by the results of the statistical analysis (Table 5). The treatment induced significant difference in all maturity stages. However, for the control samples, there were no differences between maturity stages, while for the SmartFresh^TM^−treated samples, the mature green (A) and breaker (B) groups did not differ, nor did the turning (C), pink (D) and light red (E) groups.

### 3.4. Digital Image Processing

**Figure 6:**
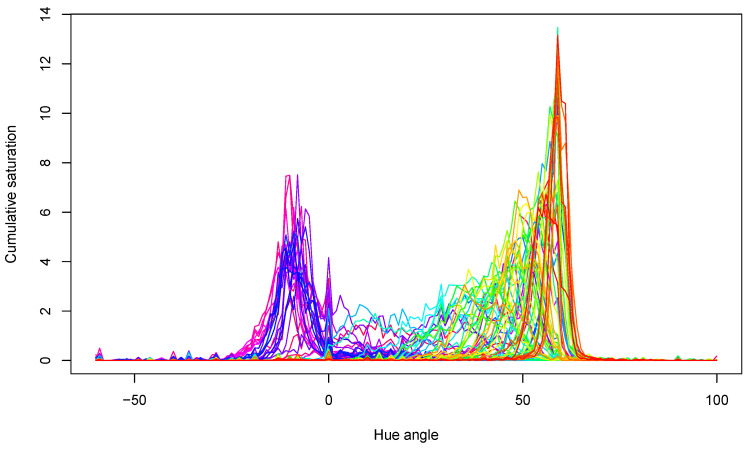
Hue spectra of all tomato samples in the experiment. Lines with different color belong to different measurements.

The acquired hue spectra for all samples are presented in Figure 6. The peaks show that samples mainly had yellow–green colors near the 60° hue angle and red colors around the 0° hue angle. Both the locations of the peaks and their values changed during storage, in agreement with the expectations.

The color change was confirmed by the statistical analysis of extracted parameters. The two-way ANOVA results are presented in Table 6. The ANOVA F values revealed that normalized color components responded more sensitively than others, especially the normalized green. Besides the main effects, significant interaction between group and 1-MCP treatment was found for normalized red and normalized green, while the interaction between 1-MCP treatment with storage time was reflected in the green, normalized red, normalized green and PQS Y coordinate parameters. These observations suggest that surface color significantly changed during the experiment, but sample groups reacted differently to the treatment. Additionally, treated and non-treated samples behaved differently during storage.

According to the analysis of correlation (Pearson) among the measured parameters, green and normalized green achieved significant and strong correlation values. The strongest correlation was obtained between the normalized red and normalized green parameters (r = −0.986, *p* < 0.01). These latter parameters showed significant correlation with PQS Y (|r| > 0.7). Based on the relationship of PQS coordinates with reference color parameters, the location of the gravity point reflected the color change as well (Table 7).

**Figure 7:**
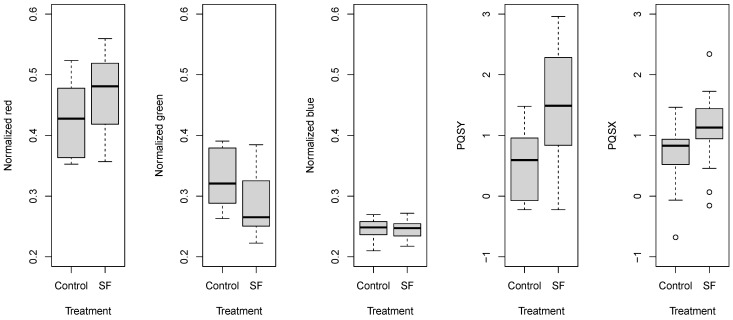
Box and whisker plots about the effect of gaseous 1-MCP treatment on normalized color parameters and PQS coordinates.

The comparison of normalized color components and PQS coordinates revealed that gaseous 1-MCP treatment induced similar changes (Figure 7). The normalized blue parameter did not show any response to the treatment. Other normalized parameters indicated that tomatoes subjected to 1-MCP treatment remained more green and less red than the control pieces. The locations of the gravity points also showed this tendency; the treated and control samples shifted. The wider range of certain parameters was attributed to the significant effect of the tomato sample group and storage time.

### 3.5. Correlation Analysis between the Different Measurement Methods

Several studies have documented the feasibility of colorimetric measurement for monitoring tomato ripening [30,31]. One of the objectives of this study was to investigate the applicability of the digital image processing for monitoring tomato ripening. The correlation analysis was performed by comparing the data obtained with the colorimeter (a*) with the DA-meter^®^ (DA-index^®^) with the acoustic firmness tester (S) and with the data obtained by digital image analysis (normalized red, normalized green, PQS coordinates). 

Naturally occurring mechanisms are typically described by a saturation curve. In the case of the DA-index^®^, which is related to chlorophyll content, there is an asymptotic curve; as the chlorophyll content decreases with maturation, the DA-index^®^ will decrease, but just as the chlorophyll content cannot take a negative value, the DA-index^®^ cannot be less than zero. The DA values will therefore approach zero as the ripening progresses. 

**Figure 8:**
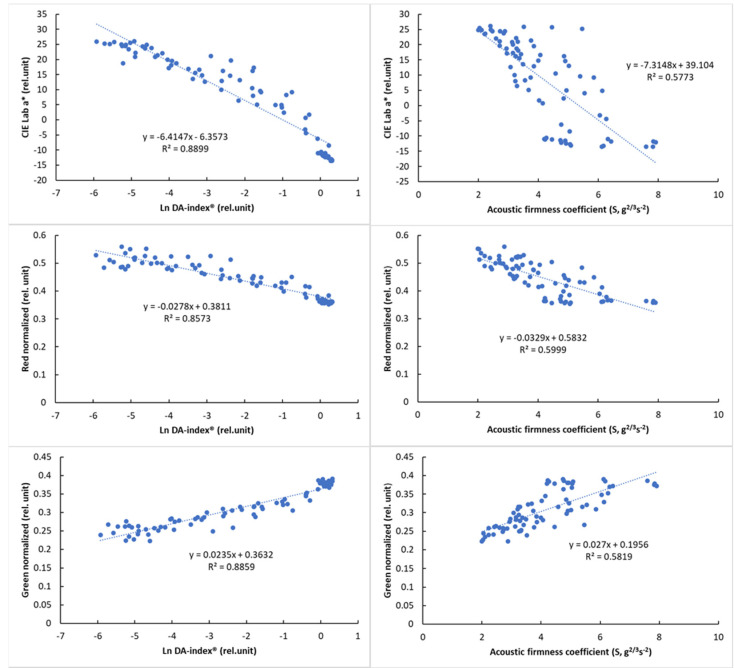

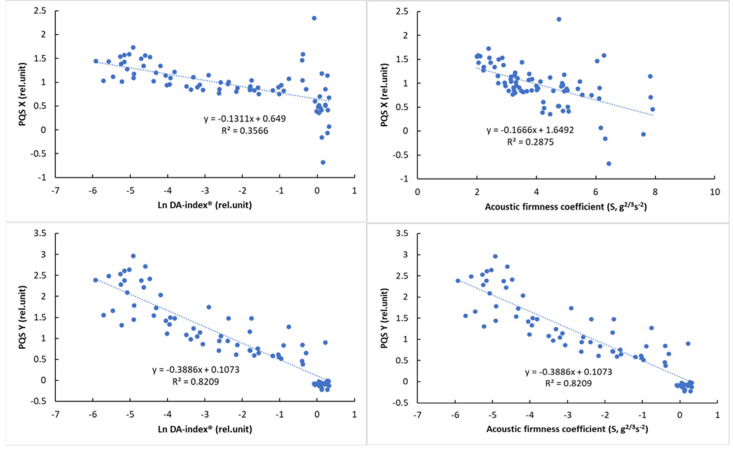
Relationship between the logarithmic DA−index^®^ and surface color parameters (**left**) and between the acoustic firmness coefficient (S) and surface color parameters (**right**).

Due to the nonlinear behavior of DA-index^®^ values, logarithmic transformation was used to present its relationship with color parameters. The relationship of the reference parameters, DA-index^®^ and stiffness, is presented in Figure 8 with visual features. 

The results of the correlation analysis (Table 8) showed a significant correlation for all parameters (*p* < 0.01). While this correlation was not so strong for PQS-X and the other parameters, a very strong correlation was observed between PQS-Y and the other parameters, especially the color parameter a* (r = 0.958, *p* < 0.01). The normalized color parameters and the a* parameter also showed a strong correlation with the other parameters, while the acoustic firmness coefficient (S) showed the strongest correlation with the PQS-Y parameter (r = −0.805), with the other parameters being less closely correlated. The observed correlation values above that of between reference parameters (DA-index^®^ and stiffness) are promising and show the potential of machine-vision-based color analysis.

## 4. Discussion

The red–green color parameter a* was relevant for our evaluation of the color measurement results, because the surface color of tomatoes changes from green to red during the transformation of the tomato skin pigments [32]. According to the CIE a*, the effect of the SmartFresh^TM^ anti-ripening treatment was most effective for mature green and breaker tomatoes. This can be explained by the fact that the rise in ethylene production reached the climacteric peak for mature samples, and ethylene, as the trigger of the ripening process, accelerated color changes and softening [33]. After the climacteric maximum, the ethylene production becomes exponential; thus, the ripening process accelerates exponentially [34]. The respiration intensity of tomatoes treated before the climacteric maximum does not increase as significantly during ripening, significantly slowing the climacteric rate [35]. A similar exponential increase in ethylene production during ripening after the inflection point and the subsequent slowing down along the saturation curve has been described for plum [36]. Furthermore, the treatment did have a positive effect on all other sample groups (turning, pink, light red and red tomatoes), which could be significantly observed after 1 week of storage time. It could be explained by the decreased ripening speed via blocking ethylene receptors and inhibiting its hormonal action by 1-MCP [2,3,4]. In general, the treatment was effective with different impacts for the sample groups. Changes in color with similar tendencies to the observation described in our study have been documented in several studies on climacteric fruits [37,38,39,40]. Moreover, softening following the same trend has also been reported [38,41].

The color change can be explained by the change in chlorophyll content, because the most typical tomato pigments are red, orange or yellow carotenoids (lycopene and β-carotene) and green chlorophyll. This is confirmed by the DA-index^®^ results, which also show that the mature green and breaker sample groups were the most affected by the SmartFresh^TM^ treatment. The turning sample group was also significantly affected by the treatment, but to a lesser extent. On the other hand, no significant effect was observed for the pink and red sample groups in terms of the DA-index^®^ as a reflection of chlorophyll content. The obtained results of the present study suggest that the measurement of the red–green color factor (a*) by reflection measurement is more sensitive to changes during ripening than the measurement of the chlorophyll content. Similar changes in the chlorophyll concentration of tomatoes have been reported in previous studies [42,43].

In the case of the acoustic firmness coefficient (S), SmartFresh^TM^ treatment induced a significant effect on each sample group. This effect was statistically significant but nominally not comparable to the effect on the red–green color factor (a*). Our observations also confirm that the mature green and breaker sample groups were most affected by the treatment, as explained above. Differences between the control and treated groups of samples became significant in each case from day 7 of storage. Thus, it can be stated that it is beneficial to use this treatment for storage longer than seven days. This may be important because previously, it has been found that tomatoes start to spoil or become unfavorable to consumers from the seventh day [44,45].

Several studies support the suitability of colorimetry for monitoring tomato ripening [30,31]. The results of digital image processing are in line with the results of color measurement and chlorophyll content measurement. During storage, the color of tomato samples changed. The effect of the treatment was primarily observed in the normalized green and normalized red values, which can also be explained by the previously described results. The method was found to be suitable for determining the ripening status of tomatoes, because the red–green color factor (a*), which reflects the ripening status, showed a strong correlation with the value of the normalized green and normalized red parameters and the PQS coordinates [37,38,39,40]. This creates the possibility of major improvement in methodology, since in the light of this knowledge, we may be able to determine the ripeness of tomatoes or other climatic fruits using a camera or even a cheaper imaging device instead of a reflectance colorimeter. The relationships determined from the results of the digital image processing method are linear functions, or saturation curves, which are most common in nature. According to these functions, the red–green color factor (a*) measured by color measurement and the DA-index^®^ can be calculated from the color parameters measured by digital image processing. With the exception of the DA-index^®^, a linear correlation between all measured and derived parameters could be detected. Strong linear correlation was found, among others, between the red–green color factor (a*) and the normalized color parameters as well as the red–green color factor (a*) and the PQS coordinates. A strong but non-linear correlation was observed between the chlorophyll-content-related DA-index^®^ and normalized color parameters as well as the DA-index^®^ and PQS coordinates. This suggests that digital image analysis is suitable for monitoring tomato ripening. 

The actual chlorophyll content of tomatoes was not determined in this series of studies (non-destructive methods were preferred); however, the relationship between total chlorophyll content and DA-index^®^ in tomatoes was previously investigated by Rahman et al. [46], and a strong linear correlation was found (r = 0.91). Nevertheless, chromatography can be used to directly determine the actual chlorophyll content for reference.

## 5. Conclusions

The presented study investigated tomato color changes in 2 weeks of refrigerated storage. The acoustic firmness and DA-index^®^ measurements were performed non-destructively; therefore, the same samples were qualified all along. The PQS method compressed hue spectra information into a 2D location, and the coordinates responded sensitively to color change. According to the observed range of colors, this analysis can be further optimized by removing indifferent hue segments. Both normalized red and green color indices and PQS coordinates were found to be able to describe tomato surface color efficiently. The results of digital image processing confirm the results of surface color measurement and chlorophyll-content-related DA-index^®^ measurement. The applied image processing method was found to be suitable for determining the ripening status of tomatoes based upon the red–green color factor (CIE a*), showing a strong correlation with the values of the machine-vision-extracted color information, providing the possibility for a major improvement in the maturity stage determination methodology of tomatoes or other climacteric fruits using a camera or even a cheaper imaging device instead of a reflectance colorimeter.

## Figures and Tables

**Table 1:** The most common non-destructive methods used to monitor tomato ripening and their advantages and disadvantages.

Technique	Advantages	Disadvantages
Colorimeter	Fast measurement. Very accurate and easy-to-access data on color.	Moderately expensive instrument. Local (point-like) data acquisition limited to the device’s optical features. In-line use is not possible.
Acoustic firmness tester	It characterizes the texture of the whole product. Fast measurement.	Only softening in the texture that can be related to ripening is measured indirectly. Other factors causing changes in texture may interfere with the accuracy of the measurement. It requires a quiet environment free of all vibrations and is not suitable for in-line measurements.
Impact firmness tester	Fast measurement; does not require expensive equipment. In-line use available.	Local (point-like) data acquisition; this may lead to differences.
Chlorophyll fluorescence measurement	The whole product can be characterized depending on the equipment’s set-up (global data acquisition and data analysis). Relatively accurate measurement related to the chlorophyll-containing green pigment content of tomatoes.	Expensive chlorophyll fluorescence imaging instruments. Some handheld or laboratory table-top devices acquire data only in point-like regions. Depending on the equipment, long measurement and complex data evaluation. The proper illumination of the product is a very important aspect, which raises questions about in-line use.
Magnetic resonance imaging (MRI)	MRI is able to distinguish physiological changes between different tissue types and physiological changes during tomato fruit ripening. It characterizes the whole product, not only point-like regions.	High investment price and running cost. Difficult data evaluation.
DA-meter^®^ (Vis-NIR meter)	Fast. Relatively cheap equipment. Easy to evaluate. It can also be used in the field/orchard/garden. NIR spectroscopy is already used in-line, so it is probably feasible for this method.	Local (point-like) data acquisition limited to the device’s optical features.
Image processing	Fast. A simple, low-cost camera is enough. The color of the whole product can be characterized; in addition, several samples can be analyzed at the same time. In-line use is possible and also can be used in the field/orchard/garden.	Skilled personnel and special data analyses are required.

**Table 2:** Maturity status of the tomatoes used in the experiment.

Maturity Status		Typical Color	Group
1	Mature green	Dark green	A
2	Breaker	Whiteish green; less than 10% of the tomato is pink	B
4	Turning	10–30% of the tomato surface is pink	C
6	Pink	30–60% of the tomato surface is pink	D
8	Light red	60–90% of the tomato surface is pink	E
12	Red	100% of the tomato surface is red; full ripeness	F

**Table 3:** Results of statistical analysis (two-way ANOVA) of a* results with means and variances at the end of the experiment (day 14).

	Treatment	
Maturity Status	Control	SF (SmartFresh^TM^)
A (mature green)	21.09 ± 3.48 ^Ba^	−11.43 ± 2.95 ^Aa^
B (breaker)	20.73 ± 2.85 ^Ba^	−11.07 ± 2.19 ^Aa^
C (turning)	23.64 ± 1.8 ^Bb^	12.63 ± 4.76 ^Ab^
D(pink)	24.83 ± 1.79 ^Bc^	18.73 ± 2.32 ^Ac^
E (light red)	25.46 ± 1.16 ^Bc^	20.89 ± 3.23 ^Ad^

Different letters indicate significantly different groups. Capital letters are used to compare treatments, while lower case letters are used to compare maturity statuses. Each group was compared using a non-parametric Games–Howell post hoc test.

**Table 4:** Results of statistical analysis (two-way ANOVA) of DA-index^®^ results with means and variances at the end of the experiment (day 14).

	Treatment	
Maturity Status	Control	SF (SmartFresh^TM^)
A (mature green)	0.055 ± 0.065 ^Ba^	1.05 ± 0.29 ^Ac^
B (breaker)	0.094 ± 0.169 ^Ba^	0.939 ± 0.246 ^Ac^
C (turning)	0.01 ± 0.019 ^Bb^	0.047 ± 0.07 ^Ab^
D (pink)	0.006 ± 0.011 ^Ab^	0.005 ± 0.011 ^Aa^
E (light red)	0.007 ± 0.011 ^Ab^	0.007 ± 0.017 ^Aa^

Different letters indicate significantly different groups. Capital letters are used to compare treatments, while lower case letters are used to compare maturity statuses. Each group was compared using a non-parametric Games–Howell post hoc test.

**Table 5:** Results of statistical analysis (two-way ANOVA) of acoustic firmness results with means and variances at the end of the experiment (day 14).

	Treatment	
Maturity Status	Control	SF (SmartFresh^TM^)
A (mature green)	2.37 ± 0.64 ^Ba^	4.2 ± 0.87 ^Ab^
B (breaker)	2.29 ± 0.62 ^Ba^	3.95 ± 0.83 ^Ab^
C (turning)	2.23 ± 0.63 ^Ba^	3.07 ± 0.95 ^Aa^
D (pink)	2.1 ± 0.56 ^Ba^	2.94 ± 1.1 ^Aa^
E (light red)	2.04 ± 0.59 ^Ba^	3.13 ± 0.75 ^Aa^

Different letters indicate significantly different groups. Capital letters are used to compare treatments, while lower case letters are used to compare maturity statuses. Each group was compared using a non-parametric Games–Howell post hoc test.

**Table 6:** Effects of group, 1-MCP treatment and storage time on color attributes according to the F value of two-way ANOVA.

Parameter	Primary Effects			Interaction Effects		
	Group (G)	1-MCP (P)	Time (T)	G × P	G × T	P × T
Red	3.64 **	0.52	6.39 *	1.59	0.44	0.03
Green	20.69 **	25.34 **	14.07 **	1.53	0.13	11.11 **
Blue	4.22 **	2.45	2.07	0.82	0.08	1.28
Red norm.	78.26 **	50.22 **	126.78 **	4.87 **	0.87	34.54 **
Green norm.	159.66 **	149.83 **	212.68 **	6.97 **	1.43	78.54 **
Blue norm.	6.28 **	0.18	22.17 **	1.42	0.40	1.18
PQS X	1.86	10.18 **	2.49	0.80	0.33	0.11
PQS Y	11.15 **	7.11 *	18.18 **	1.06	0.08	8.70 **

Significant F values are marked with ** *p* < 0.01, * *p* < 0.05.

**Table 7:** Correlation matrix of measured parameters (Pearson’s correlation).

	Green	Blue	R Norm	G Norm	B Norm	PQS X	PQS Y
Red	0.124	0.449 **	0.336 **	−0.347 **	0.209	0.147	0.238 *
Green		0.883 **	−0.874 **	0.866 **	0.681 **	−0.275 *	−0.643 **
Blue			−0.615 **	0.560 **	0.662 **	−0.118	−0.394 **
Red norm.				−0.986 **	0.792 **	0.344 **	0.724 **
Green norm.					0.681 **	−0.355 **	−0.746 **
Blue norm.						−0.219	−0.458 **
PQS X							0.192

Significant correlations are marked with ** *p* < 0.01, * *p* < 0.05.

**Table 8:** Correlation matrix of measured parameters (Spearman’s correlation).

	DA-Index^®^	a*	Red Norm.	Green Norm.	PQS-X	PQS-Y
S	0.762	−0.782	−0.783	0.787	−0.555	−0.805
DA-index^®^		−0.967	−0.911	0.929	−0.640	−0.904
a*			0.935	−0.962	0.692	0.958
Red norm.				−0.969	0.644	0.911
Green norm.					−0.693	−0.948
PQS-X						0.706

All correlations were significant at *p* < 0.01.

## Data Availability

The data presented in this study are available on request from the corresponding authors.
